# Emotional Reactivity and Emotion Regulation Among Young Adults During COVID-19 Lockdown: The Moderating Role of Gender and Engagement in Sports

**DOI:** 10.3389/fpsyg.2021.774732

**Published:** 2021-10-26

**Authors:** Marijana Mladenović, Nikola Stojanović, Darko Stojanović, Mladen Živković, Dragana Aleksić, Gorana Tešanović, Vladimir Momčilović

**Affiliations:** ^1^College of Sports and Health, University of Belgrade, Belgrade, Serbia; ^2^Faculty of Sport and Physical Education, University of Niš, Niš, Serbia; ^3^Pedagogical Faculty in Vranje, University of Niš, Vranje, Serbia; ^4^Faculty of Sport and Physical Education, University of Priština-Kosovska Mitrovica, Leposavić, Serbia; ^5^Faculty of Physical Education and Sport, University of Banja Luka, Banja Luka, Bosnia and Herzegovina

**Keywords:** emotion regulation, emotional reactivity, COVID-19, lockdown, gender differences, physical activity, sport

## Abstract

The effects of the COVID-19 pandemic on mental health have not been fully inspected among the young adults’ population. The objectives of the present study were: (1) to examine differences in emotional reactivity and emotion regulation between, both gender and sports engagement level during the first 2 weeks of the lockdown; and (2) to examine the possible impact of emotion regulation on emotional reactivity, and possible significant roles of gender and sports engagement level as moderators. This cross-sectional study included 315 Serbian young adults (aged 18–26 years old) during COVID-19 lockdown. Respondents answered socio-demographic questions and the Serbian version of the Multidimensional Emotion Questionnaire (MEQ). The results of confirmatory factor analysis indicated good fit for both positive and negative reactivity scales (SRMR = 0.037; CFI = 0.984, RMSEA = 0.046, and SRMR = 0.055; CFI = 0.964, RMSEA = 0.064, respectively). Gender differences were found in both positive (*p* = 0.039; *d* = 0.28) and negative emotional reactivity scales (*p* < 0.001; *d* = 0.60), with females reported lower and higher values, respectively. Professional athletes presented higher scores in positive reactivity scale in comparison to non-athletes (*p* < 0.001; *d* = 0.78) and recreational athletes (*p* = 0.034; *d* = 0.34) during 2 weeks of COVID-19 lockdown. Conversely, professional athletes scored lower in negative emotional reactivity scale in comparison to non-athletes (*p* < 0.001; *d* = 0.85) and recreational athletes (*p* = 0.006; *d* = 0.42). Both gender and sports engagement level differences were found for negative, but not for positive emotion regulation scale. Furthermore, results showed that engagement in sports level plays a significant role as moderator in relationship between negative regulation and negative reactivity, where professional athletes presented significant interaction effect and predicted lower negative reactivity scores compared to non-athletes and recreational athletes. However, gender does not moderate the influence of emotion regulation on emotional reactivity either positive or negative. Engagement in sports as a lifestyle may contribute to better emotional harmony especially in the crisis situation as COVID-19 lockdown.

## Introduction

The COVID-19 pandemic as a global health crisis has affected not only general health but also everyday life (Restubog et al., 2020). Despite the belief that they could not potentially develop serious symptoms of the disease, young adults in Serbia were aware of the seriousness of the public health situation. The importance of physical distancing has been enlarged by the influence of national health experts and government officials through television broadcasting. Thus, information about a potential epidemiological catastrophe could influence the development of protective patterns, which may result in increased physical distancing and self-isolation (Cvetković et al., 2020). Disease outbreaks not only negatively affect daily activities but can also cause acute and long-term negative effects on overall well-being (Holmes et al., 2020). Although, it is well known that engagement in sport or physical activity has many benefits on psychological well-being, imposed social distancing measures led to significant decrease in physical activity levels (Blazevic et al., 2021). Therefore, the consequences are not only economical and physical but can also affect mental health (Restubog et al., 2020). The negative psychological consequences that people encounter the most are anxiety (Hyland et al., 2020), psychological distress and depression, and reduced quality of life (Šakan et al., 2020). These emotional responses are adaptive following a crisis, as long as the ability to live a balanced life remains intact (Panayiotou et al., 2021). However, some evidence suggests that the COVID-19 pandemic has a negative impact on the overall quality of life (Solomou and Constantinidou, 2020). Recently, Zovko and Budler (2021) emphasized that moderate physical activity is one of the best stress management methods and recommended that all healthy individuals should practice moderate-intensity exercise of all types during pandemic crisis. Emotion regulation is an important factor that could influence the extent of the negative impact of a pandemic on overall well-being and involves the conscious or unconscious efforts to influence the experience, expression, duration, and magnitude of emotions (Gross, 2002, 2015). However, although different constructs, emotion regulation cannot be separated from emotional reactivity. Emotional reactivity refers to the processes that determine the nature and strength of an individual’s unaltered emotional response (Gross, 2002). According to Klonsky et al. (2019), emotional reactivity consists of sensitivity, intensity, and persistence of the emotional response. Sensitivity refers to the magnitude of stimulus required to induce an emotional response, and determine how frequently an emotional response is triggered. Intensity refers to the magnitude of the emotional response when it occurs. Persistence refers to how long the emotional response lasts before recovery to baseline. Moreover, Tracy et al. (2014) argue that increased reactivity could be explained in terms of decreased regulation, and vice versa. Recently, Klonsky et al. (2019) developed the Multidimensional Emotion Questionnaire (MEQ), a single inventory to assess both emotional reactivity and emotion regulation. However, it remains unclear whether MEQ could potentially differentiate emotional reactivity and emotion regulation regarding gender. Some studies emphasize that there are differences in the emotional reactivity and emotion regulation between females and males (Gross and John, 2003; Mackiewicz et al., 2006; Nitschke et al., 2006; McRae et al., 2008; Domes et al., 2010; Gardener et al., 2013; Zimmermann and Iwanski, 2014; Costa et al., 2020), while others failed to support this statement (Gross, 2002, 2015; Domes et al., 2010; Lane et al., 2011). A possible explanation for such diversity in emotional responding could be a function of two dissociable processes: emotional reactivity and emotion regulation (McRae et al., 2008). If so, gender or engagement in sport differences in emotional responding could arise either from differences of actual emotional reactivity, or from differences in how those emotions are regulated, or either both. Based on previous statements, gender differences in emotion regulation and emotional reactivity requires further explanation. On the other hand, several studies confirmed that athletes have higher dispositional hope (Curry et al., 1997), optimism (Nicholls et al., 2008), perseverance (Laborde et al., 2014, 2016), resilience (Padesky and Mooney, 2012), and adaptive emotion regulation strategies (Lane et al., 2009, 2011; Laborde et al., 2014; Doorley and Kashdan, 2021). Furthermore, it has been reported that professional athletes had better mental health status than non-athletes (Şenışık et al., 2021) and showed lower negative emotional state values than expected average (Leguizamo et al., 2021) during COVID-19 lockdown. It should be noted, there were no previous studies to confirm if MEQ could differentiate emotion regulation and emotional reactivity based on engagement in sports (non-athletes, recreational athletes, and professional athletes). On this account, there is a gap in the literature in which different approaches could allow potential contribution to further explain emotion regulation and emotional reactivity and their interaction. Moreover, the interaction between emotion regulation and emotional reactivity could be moderated by both gender and engagement in sports. To our best knowledge, there were no previous studies that assessed such moderating role of both gender and engagement in sports during COVID-19 lockdown. Therefore, the objectives of the present study were: (1) to examine differences in emotional reactivity and emotion regulation between both gender and engagement in sports during the first 2 weeks of the lockdown; and (2) to examine the possible interaction between emotion regulation on emotional reactivity, and whether there is the significant role of both gender and engagement in sports as moderators.

## Materials and Methods

### Study Design and Procedures

This cross-sectional study was conducted 2 weeks after the lockdown imposed by the Republic of Serbia Government on March 15, 2020. The present study included young adults and employed a self-reported questionnaire assessing emotional reactivity and emotion regulation during COVID-19 lockdown. The questionnaire was adapted in Google forms, and the link was disseminated to the targeted population. To avoid duplications, respondents could provide only one response per Google account. The completion of the questionnaire was not limited by time. To ensure the complete honesty of the self-reported emotional reactivity and emotion regulation, respondents were informed that their answers would remain anonymous, and the results would be used only for research purposes. Participants who did not respond received an email reminder with a personalized link to the respective survey. The emails were sent at random time points throughout the day. Incompletely administered responses with ambiguous outcomes were not included in the further analysis. 315 responses out of 317 met the inclusion criteria for further analysis. The questionnaire was preceded by sociodemographic questions. Therefore, it was possible to examine differences and relationships between different categories (gender and engagement in sports). The procedures in this study were conducted according to the Declaration of Helsinki as a statement of ethical principle for research involving human subjects.

### Participants

The sample was comprised of a total of 315 respondents, ranging from 18 to 26 years of age, from which 240 (76.2%) were females, and 75 (23.8%) were males. We should note, since this research was part of a more extensive research and data collection, the exact age of the respondents was not collected. The respondents had the option to choose which age range they belong to (18–26, 27–33, 34–39, 40–49, 50–65). Furthermore, respondents reported their engagement in sports, from which 65 (20.6%) were non-athletes, 159 (50.5%) were recreational athletes, and 91 (28.9%) were professional athletes. Within the e-mail of the attached questionnaire, the respondents were fully acquainted with the research procedure and informed that they could withdraw from the study at any time.

### Measures

Multidimensional emotion questionnaire (MEQ). The MEQ (Klonsky et al., 2019) was used in the present study. The MEQ assesses five positive (happy, excited, enthusiastic, proud, and inspired) and five negative emotions (sad, afraid, angry, ashamed, and anxious). This questionnaire is composed of four types of emotional scales: (1) 10 discrete emotions, (2) three subcomponents of emotional reactivity (frequency, intensity, and persistence), (3) superordinate dimensions of emotional reactivity (positive and negative), and (4) regulation. Discrete emotions are not further presented in this study. However, relevant items for discrete emotions were used to form composite scores included in the further analysis. Subcomponents of emotional reactivity were calculated for positive frequency, positive intensity, positive persistence, negative frequency, negative intensity, and negative persistence by summing scores for the specific items. For example, the negative persistence subscale is formed by summing the persistence scores for sad, afraid, angry, ashamed, and anxious. Superordinate positive and negative emotionality scales, frequency, intensity, and persistence scores for positive emotions were summed to form an overall positive emotional reactivity score, and frequency, intensity, and persistence scores for negative emotions are summed to form an overall negative emotional reactivity score. Emotion regulation scores were calculated for positive emotion regulation and negative emotion regulation by summing scores for the specific items.

Statements were evaluated using a four-point Likert scale. The response options for each question were as follows: (1) How Often (about once each month, about once each week, about once each day, about 2–3 times each day, more than 3 times each day), (2) How Intense (very low, low, moderate, high, very high), (3) How Long-Lasting (less than 1 min, 1–10 min, 11–60 min, 1–4 h, longer than 4 h), and (4) How Easy to Regulate (very easy, easy, moderate, difficult, and very difficult).

For the purposes of this research, the Serbian version of the previously described inventory was created and employed. Therefore, it was necessary to evaluate the fit of the model for overall negative and positive emotion scales. The results of confirmatory factor analysis indicated good fit for both positive and negative reactivity scales (SRMR = 0.037; CFI = 0.984, RMSEA = 0.046, and SRMR = 0.055; CFI = 0.964, RMSEA = 0.064, respectively). This result is consistent with one provided in the original inventory (Klonsky et al., 2019). Internal consistency in this study for all scales and subscales proved to be good (median = 0.85; range 0.76–0.92).

### Data Analysis

All data analyses were carried out using IBM SPSS Statistics (Version 23.0). Descriptive statistics were computed for all sociodemographic and study variables. Means, medians, standard deviations, frequencies, percentages, and Pearson’s bivariate correlations where appropriate were computed to describe both categorical and continuous variables for the total sample. Confirmatory factor analysis (CFA) was performed to evaluate the fit for the Serbian version of MEQ items indexing both overall positive and negative emotions scales. Independent samples *t*-test was performed to assess the differences between gender (female vs. male) in continuous variables, and One-way ANOVA with Bonferroni *post hoc* analysis for the differences between groups of different levels of engagement in sports (non-athletes, recreational athletes, and professional athletes) during COVID-19 lockdown. Cohen’s *d* analyses were performed to evaluate the effect size. According to Cohen’s guidelines (Cohen, 1992), effect size was interpreted as small (*d* = 0.2), medium (*d* = 0.5), and large (*d* = 0.8). A multiple moderation model was performed to examine if the relationship between emotion regulation and overall emotional reactivity was moderated by both gender and engagement in sports. The moderation effect was estimated using SPSS macro PROCESS (Model 2) for moderation based analysis (Hayes, 2017). Emotion regulation was used as the independent variable and overall emotional reactivity as the dependent variable. A bootstrapping procedure was used (with 10,000 resamples) in moderation based analysis. Significance was set at the 0.05 level.

## Results

Demographic characteristics of the sample are presented in Table 1.

**TABLE 1:** Summary of demographic data (frequencies and percent).

	*n* (%)
**Gender**	
Men	75 (23.8)
Women	240 (76.2)
**Engagement in sports**	
Non-athletes	65 (20.6)
Recreational athletes	159 (50.5)
Professional athletes	91 (28.9)
**PA during COVID-19 lockdown**	
*Non-athletes*	
No PA	44 (67.7)
2–3 times per week	13 (20)
Once per day	8 (12.3)
*Recreational athletes*	
No PA	32 (20.1)
2–3 times per week	81 (50.9)
Once per day	46 (28.9)
*Professional athletes*	
No PA	16 (17.6)
2–3 times per week	41 (45.1)
Once per day	34 (37.4)
**Living status during COVID-19 lockdown**	
Single	20 (6.3)
With partner	6 (1.9)
With family members	289 (91.7)

Gender-associated variation showed significant differences among groups in negative frequency, intensity, and persistence subscales, as well as overall negative emotional reactivity and negative emotion regulation scales (see Table 2), with females presenting higher scores compared to males. Conversely, males presented significantly higher scores in positive frequency, intensity, and overall positive emotional reactivity. Small to moderate effects regarding gender were present in both positive and negative emotion regulation and emotional reactivity scales.

**TABLE 2:** Gender differences in positive and negative emotional reactivity and emotional regulation scales, Independent Samples Test.

	Male (*n* = 75)	Female (*n* = 240)	*P* (2-tailed)	Cohen‘s d
Positive frequency	16.16 ± 4.19	14.53 ± 4.30	0.004	0.38
Positive intensity	17.25 ± 4.11	15.78 ± 4.56	0.013	0.33
Positive persistence	14.37 ± 4.96	14.22 ± 4.64	0.840	0.03
Overall positive	47.76 ± 11.51	44.53 ± 11.87	0.037	0.27
Positive regulation	9.4 ± 4.61	8.99 ± 3.58	0.484	0.11

	**Male (*n* = 75)**	**Female (*n* = 240)**	***P* (2-tailed)**	**Cohen‘s d**

Negative frequency	9.45 ± 3.39	11.49 ± 4.27	<0.001	0.50
Negative intensity	11.61 ± 3.56	13.23 ± 4.41	0.002	0.38
Negative persistence	10.09 ± 3.37	12.38 ± 3.96	<0.001	0.60
Overall negative	31.16 ± 8.63	37.09 ± 10.96	<0.001	0.57
Negative regulation	10.85 ± 4.09	13.90 ± 4.83	<0.001	0.65

Variation associated with the engagement in sports showed significant differences among groups in negative emotion regulation and emotional reactivity (overall positive and negative) (see Table 3). Recreational athletes (Group 2) and professional athletes (Group 3) presented lower scores compared to non-athletes (Group 1) in both overall negative emotion regulation (Group 1 vs. Group 2, *d* = 0.41; Group 1 vs. Group 3, *d* = 0.82) and negative emotional reactivity scales (Group 1 vs. Group 2, *d* = 0.34; Group 1 vs. Group 3, *d* = 0.80) The professional athletes also presented lower scores compared to recreational athletes in both overall negative emotion regulation (Group 2 vs. Group 3, *d* = 0.36) and negative emotional reactivity scales (Group 2 vs. Group 3, *d* = 0.42). Furthermore, recreational athletes and professional athletes presented higher scores on the overall positive emotional reactivity scale compared to non-athletes (Group 1 vs. Group 2, *d* = 0.45; Group 1 vs. Group 3, *d* = 0.78). The professional athletes also presented higher scores in the overall positive emotional reactivity scale compared to recreational athletes (Group 2 vs. Group 3, *d* = 0.34). Interestingly, non-athletes presented higher scores of positive emotion regulation compared to recreational and professional athletes but non-significant.

**TABLE 3:** Differences in positive and negative emotional reactivity and positive and negative emotion regulation between three levels of engagement in sport.

	Non-athletes (*n* = 69) Group 1 Mean ± SD	Recreational athletes (*n* = 159) Group 2 Mean ± SD	Professional athletes (*n* = 91) Group 3 Mean ± SD	F	*Post-hoc*
					1 < 2**
OP	40.06 ± 12.26	45.26 ± 11.29	49.10 ± 11.162	11.80**	1 < 3**
					2 < 3*
					1 > 2*
ON	39.94 ± 11.80	36.11 ± 10.77	31.88 ± 8.48	11.68**	1 > 3**
					2 > 3**
					1 > 2
PR	9.28 ± 3.86	9.01 ± 3.73	9.09 ± 4.08	0.11	1 > 3
					2 < 3
					1 > 2*
NR	15.23 ± 4.58	13.25 ± 4.90	11.57 ± 4.33	11.66**	1 > 3**
					2 > 3*

*OP*, *Overall Positive; ON*, Overall Negative; PR, Positive Regulation; NR, Negative regulation; * and ** indicates statistical significance at alpha levels of 0.05 and 0.01, respectively.

We computed internal consistencies, intercorrelations, means, and standard deviations for the emotional reactivity subscales (see Table 4). Internal consistency for the emotional reactivity subscales ranged from good to very good, with a low of 0.76 for negative persistence and a high of 0.88 for positive regulation. Internal consistencies for both overall positive and negative scales were excellent (0.92 and 0.90). Intercorrelations for positive reactivity scales (median = 0.73; range 0.55–0.86) and among negative reactivity scales (median = 0.70; range 0.55–0.87) were positive and from medium to large magnitude. Mean scores are also reported in Table 4. However, it is important to note that means for different types of reactivity subscales are not comparable because the scales had different labels to reflect subscale content.

**TABLE 4:** MEQ Emotional Reactivity and Emotional Regulation scales (intercorrelations, descriptive statistics, and coefficient alpha).

Scale	PF	PI	PP	NF	NI	NP	PR	NR	OP	ON	Mean (*SD*)	Alpha
PF	−	0.73	0.55	−0.25	−0.25	−0.30	−0.20	−0.35	0.86	−0.31	14.92 (4.33)	0.81
PI		−	0.67	−0.41	−0.15	−0.21	−0.09^NS^	−0.28	0.91	−0.30	16.13 (4.49)	0.85
PP			−	−0.41	−0.29	0.03^NS^	−0.13*	−0.21	0.85	−0.26	14.25 (4.71)	0.85
NF				−	0.70	0.55	0.15*	0.54	−0.41	0.87	11.01 (4.17)	0.79
NI					−	0.64	0.21	0.59	−0.26	0.90	12.84 (4.27)	0.78
NP						−	0.16	0.60	−0.18	0.83	11.83 (3.94)	0.76
PR							−	0.36	−0.16	0.20	9.09 (3.85)	0.88
NR								−	−0.32	0.67	13.17 (4.83)	0.86
OP									−	−0.33	45.40 (11.85)	0.92
ON										−	35.68 (10.74)	0.90

*PF, Positive Frequency; PI, Positive Intensity; PP, Positive Persistence; NF, Negative Frequency; NI, Negative Intensity; NP, Negative Persistence; PR, Positive Regulation; NR, Negative Regulation; OP, Overall Positive; ON, Overall Negative.*

*All correlations are statistically significant at alpha level of 0.01, except where ^∗^ indicates significant correlations at alpha level of 0.05 and ^*NS*^ indicates non-significant correlations.*

Emotion regulation, gender, and engagement in sports were used to predict emotional reactivity. Data were checked for outliers and assumptions of regression, and no violations were found. The PROCESS macro (Hayes, 2017) was used to center variables, and analyze the interaction between emotion regulation and emotional reactivity.

**FIGURE 1:**
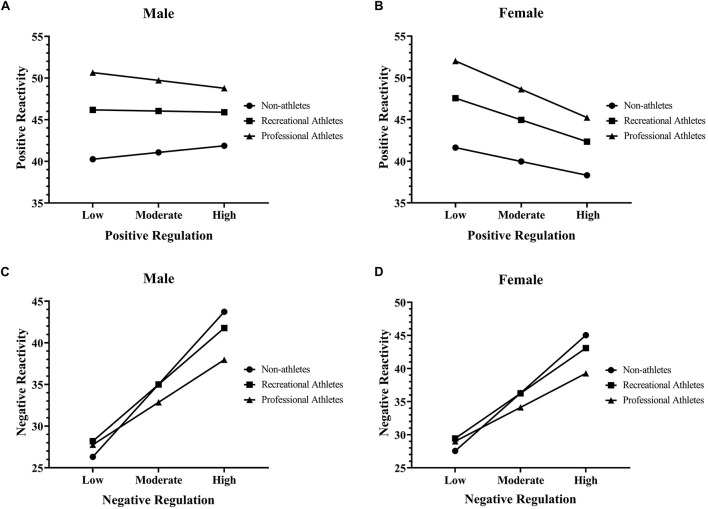
The association between positive emotional regulation and emotional reactivity moderated by gender and engagement in sports **(A,B)**. The association between negative emotional regulation and emotional reactivity moderated by gender and engagement in sports **(C,D)**.

The overall model of association between positive emotion regulation and overall positive emotional reactivity moderated by gender, and engagement in sports was significant, [*F*_(7,307)_ = 5.11, *p* < 0.001, *R*^2^ = 0.10]. Test of the highest unconditional order interactions showed that the moderation of the effect of positive regulation by both gender (see Figure 1) and engagement in sports (see Figure 1) was not significant [*F*_(1, 307)_ = 2.94, *p* = 0.088; *F*_(2,307)_ = 0.41, *p* = 0.661], and uniquely accounts for 0.9 and 0.2% of the variance, respectively. However, it is apparent both from the estimate of positive regulation and gender interaction and conditional effect, that the effect of positive regulation on overall positive reactivity is in opposite direction larger for females than males. For female recreational athletes, there was a significant decrease in overall positive emotional reactivity score when emotion regulation score increased by one unit *b* = −0.68, *t* = −2.55, *p* = 0.011. Interestingly, female athletes showed the largest decrease in positive emotional reactivity scores when positive regulation scores are increased by one unit, *b* = −0.88, *t* = −2.47, *p* = 0.014.

The overall model of association between negative emotion regulation and overall negative emotional reactivity moderated by gender, and engagement in sports was significant, [*F*_(7, 307)_ = 38.04, *p* < 0.001, *R*^2^ = 0.46]. Test of the highest unconditional order interactions showed that the moderation of the effect of negative emotion regulation by engagement in sports was significant [*F*_(2, 307)_ = 3.07, *p* = 0.048], and uniquely accounts for 1.1% of the variance. Conversely, moderating effect of gender was not significant [*F*_(2, 307)_ = 0.0006, *p* = 0.980] (see Figure 1). Furthermore, it is apparent both from the estimate of negative regulation and engagement in sports interaction and conditional effect, that the effect of negative regulation on overall negative emotional reactivity is somewhat larger for non-athletes in comparison with recreational athletes and professional athletes. For female non-athletes and recreational athletes, there was a significant increase in overall negative emotional reactivity score when negative emotion regulation score is increased by one unit, *b* = 1.81, *t* = 8.27, *p* < 0.01 and *b* = 1.41, *t* = 10.52, *p* < 0.01, respectively. Female professional athletes showed the lowest increase in overall negative emotional reactivity scores when negative regulation scores increase by one unit, *b* = 1.06, *t* = 4.80, *p* < 0.01 (see Figure 1). For male non-athletes and recreational athletes, there was a significant increase in overall negative emotional reactivity scores when negative emotion regulation scores increase by one unit *b* = 1.80, *t* = 5.63, *p <* 0.01 and *b* = 1.41, *t* = 5.328, *p <* 0.01, respectively. Male professional athletes showed the lowest increase in overall negative emotional scores when negative regulation scores are increased by one unit, *b* = 1.06, *t* = 4.14, *p* < 0.01 (see Figure 1).

## Discussion

The aim of this study was to evaluate the level of emotion regulation and emotional reactivity considering gender, engagement in sports level (non-athletes, recreational athletes, and professional athletes) in a sample of Serbian young adults during COVID-19 lockdown, and to inspect the moderating role of both gender and engagement in sports in the association between emotion regulation and emotional reactivity. Females presented lower values in positive frequency, positive intensity, and overall positive reactivity scores. Conversely, females presented higher values in negative frequency, negative intensity, negative persistence, and overall negative scores. Individuals who were classified as athletes presented lower scores in both negative emotion regulation and emotional reactivity scores, and higher scores in positive emotional reactivity subscale compared to individuals classified as non-athletes and recreational athletes. However, there were no differences between groups in positive emotion regulation scores. Moreover, lower levels of positive emotion regulation (higher scores) were negatively associated with overall positive emotional reactivity subscale, meaning that individuals with lower positive emotion regulation capabilities experience lower positive emotional reactivity. However, this association was not significantly moderated by both gender and engagement in sports level. Conversely, lower levels of negative emotion regulation (higher scores) were positively associated with overall negative emotional reactivity, meaning that individuals with lower negative emotion regulation capabilities experience higher negative emotional reactivity. This association was moderated by engagement in sports level, but not by gender.

Present study found significant differences across gender (see Table 2). Possible explanation for eventual differences could be that females are more likely to expect negative events regarding COVID-19 lockdown. These gender differences in emotion regulation strategies may be present due to the fact that females tend to express their emotions more than males (Gross and John, 2003), and this period of social isolation might have been an obstacle to this expressivity (Costa et al., 2020). Furthermore, previous studies have shown that anticipation of negative stimuli evokes emotional responses which are related to increased amygdala activity in females (Mackiewicz et al., 2006; Nitschke et al., 2006; McRae et al., 2008; Gardener et al., 2013). Domes et al. (2010) argue that women might attempt to downregulate their emotions as soon as the negative stimuli appeared. Altogether, findings from above mentioned studies suggest that women have greater early emotional reactivity to negative stimuli, thus supporting a female negativity bias. However, initially enhanced emotional response in females could influence these attempts to be less effective in overall emotion regulation. These findings are consistent with the results of our study. Zimmermann and Iwanski (2014) argue that females report more social support seeking and dysfunctional rumination and males report more suppression, avoidance, and passivity. Apparently, physical distancing during COVID-19 lockdown could potentially have a greater negative impact on females, therefore present results are not surprising. However, we should be fully aware that making generalized statements, could potentially mask the real picture. For example, when we explore the moderating influence of gender on negative emotional reactivity, it is obvious that the conditional effects of the focal predictor at values of the moderator are very similar for female and male athletes in the present study (females: *b* = 1.06; males = *b* = 1.06). Similar trend could be seen across non-athletes (females: *b* = 1.81; males = *b* = 1.80) and recreational athletes (females: *b* = 1.41; males = *b* = 1.41). It seems that gender does not have a significant moderating effect in predicting the overall negative emotional reactivity. We argue that the research on gender differences in behavioral and neural responding to emotional stimuli is inconsistent, and it is not possible to draw a unanimous conclusion regarding this matter. One reason for the observed inconsistencies might be the fact that most studies could not differentiate between emotional reactivity and emotion regulation (Domes et al., 2010). We can support previous statement with the findings in our study. Although, the positive regulation scale demonstrated incremental prediction of a measure of emotion dysregulation beyond the MEQ’s overall positive and negative emotional reactivity scales, negative regulation scale did not entirely. The negative regulation scale does indeed relate to emotion dysregulation, however, it correlated 0.69 with the negative reactivity scale, suggesting certain difficulty in distinguishing these constructs. These findings are consistent with the results of a recent study (Klonsky et al., 2019). Moreover, emotional responding has been conceptualized as interaction between emotional reactivity and emotion regulation, including reappraisal and suppression (Gross, 2002, 2015). It is possible that gender differences might be related to enhanced emotional reactivity and reduced emotion regulation regarding reappraisal. However, our study could not support these findings. As previously stated, although there were significant differences between males and females in our study (see Table 2), there was no interaction effect between negative emotional reactivity and emotion regulation moderated by gender (see Figure 1). It seems, that interaction effect among same entities (male vs. female non-athletes; male vs. female recreational athletes; male vs. female athletes) is not present based on the results of our study. Our results are consistent with the findings of a study conducted by Lane et al. (2011). These authors examined if there are any differences in emotion regulation between female and male athletes, and there were no significant findings to support that statement.

However, engagement in sport levels could have a valuable moderating effect. We argue that it is important that engagement in sports should be perceived as an important area for studying emotion, particularly as emotion regulation influence the daily life of athletes beyond training and competition. It is proposed that practitioners and researchers can identify moderating factors that could influence emotion regulation (Lane et al., 2011). Results of the present study showed that negative emotion regulation can predict negative emotional reactivity, and the moderating effect of engagement in sports is rather significant. This is very important in the context of physical distancing and self-isolation during pandemic outbreak like COVID-19. Zimmermann and Iwanski (2014) argue that the intensity and quality of the reported emotions are associated with the use of specific emotion regulation strategies, where the differential preferences of emotion regulation might be an indicator of emotion-specific activation and functionality of emotion regulation strategies. There are a few possible explanations why athletes scored better on both overall positive and negative reactivity subscales, and negative emotion regulation subscale. Athletes potentially have higher dispositional hope than non-athletes, which could impact overall performance (Curry et al., 1997). Moreover, participants involved in sports could be more optimistic (Nicholls et al., 2008), and athletes may develop perseverance in order to adjust to a specific environment (Guillén and Laborde, 2014; Laborde et al., 2016). Nonetheless, it seems possible that sport could develop resilience (Padesky and Mooney, 2012), and may provide the specific environment to develop this trait, because sport commonly confronts the athlete with unpleasant events. Costa et al. (2020) argued that athletes could potentially have more adaptive emotion regulation strategies to overcome negative emotions during COVID-19 lockdown. These regulatory strategies such as reappraisal and acceptance are explored less frequently among athletes in favor of studies focused on sport-specific coping and the effects of regulatory strategies on athletic performance (Lane et al., 2009, 2011; Laborde et al., 2014; Doorley and Kashdan, 2021). Athletes also have ability to improve and maintain savoring. Doorley and Kashdan (2021) argue that athletes and coaches are mainly focused on overcoming negative emotions in response to negative events than upregulating positive one. Authors emphasize that interventions like enhancing character strengths, gratitude, savoring, and compassion not only enhance positive emotions, but facilitate healthy responses to stress. However, the effectiveness of various emotion regulation strategies related to both positive and negative events in daily life, could be sport or non-sport related. Therefore, this matter should be explored in more detail.

There are several important study limitations and future directions. Although, the results of the present study showed that there is significant moderation role of engagement in sports, but the overall effect is rather small, therefore this issue should be investigated more thoroughly.

Being engaged in physical activity and regular exercise during COVID-19 lockdown may have attenuated the differences amongst individuals of different level of engagement in sports (non-athletes, recreational athletes, and professional athletes). It is hypothesized that regular physical activity and exercise have been related to numerous mental benefits, including improved mental well-being (Mandolesi et al., 2018; Frontini et al., 2021; Sokić et al., 2021). The results from the demographic data in our study indicate that professional athletes were engaged in more frequent PA and exercise (see Table 1). However, these results cannot provide credible evidence, because the self-reported measures of physical activity in presented form is highly speculative and unreliable. Therefore, this variable was not included in the further analysis. Moreover, the present study did not examine the discrepancy between optimal emotion regulation and emotional reactivity relative to COVID-19 lockdown conditions. Therefore, we were not able to find cause-and-effect about the influence of lockdown on emotion regulation and emotional reactivity. Future studies should explore changes in optimal emotion regulation and emotional reactivity and individual’s coping strategies to upregulate and down-regulate emotions during COVID-19 lockdown. For example, if current emotional intensity is lower than optimal, than it should be upp-regulated, possibly by using strategies like reappraisal to decrease negative and increase positive emotions (Gross, 2002). Therefore, it is possible to examine whether the interaction between emotion regulation and emotional reactivity is moderated by engagement in sports and gender or related to the individual’s ability to overcome negative emotions regardless of engagement in sports and gender.

Finally, we should point out that the most of our respondents were surrounded with their family members (91.7%) during COVID-19 lockdown. Holmes et al. (2020) argue that physical isolation could cause unpleasant emotions which could be associated with altered stress, anxiety, and depression. However, the physical isolation could be compensated by altering positive family interactions, and therefore generating resilience to diminish the stressful circumstances (Holmes et al., 2020). This is important to note, because it is possible that emotion regulation strategies in conditions of self-isolation could be different, and more dependent on one’s own ability to regulate emotions. Therefore, interaction between emotion regulation and emotional reactivity moderated by engagement in sports level during pandemic outbreak, could not entirely be explained based on data provided in the present study. Van Bavel et al. (2020) argue that term “social distancing” should be replaced with “physical distancing” because social interaction could be maintained even when individuals are physically separated. This statement should be highly considered before drawing any decisive conclusions.

## Conclusion

In order to explore interaction between emotion regulation and emotional reactivity it was necessary to examine are there any differences regarding gender and engagement in sports, or any possible moderating effect of both gender and engagement in sports. Based on findings in the present study we were able to support that female individuals tend to present higher scores in both negative emotion regulation and emotional reactivity subscales. Moreover, it seems that professional athletes have a greater capability to regulate negative emotions than non-athletes and recreational athletes. Furthermore, results of our study proved that engagement in sports has a significant, yet small moderating effect, where professional athletes presented significant interaction effect and predicted lower negative reactivity scores compared to non-athletes and recreational athletes. However, based on our findings, gender does not moderate the relationship between either positive or negative emotion regulation on emotional reactivity, therefore this matter should be explored in more detail. We emphasize that engagement in sports as a lifestyle can contribute to better emotional harmony especially in the crisis situation as COVID-19 lockdown. The present study may contribute to improve mental health by stressing the importance of engagement in sports during COVID-19. However, more research on this matter is required. Future studies should explore daily changes in optimal emotional reactivity and emotion regulation and individual‘s coping strategies to upp-regulate and down-regulate emotions during COVID-19 lockdown.

## Data Availability Statement

The raw data supporting the conclusions of this article will be made available by the authors, without undue reservation.

## Ethics Statement

Ethical review and approval was not required for the study on human participants in accordance with the local legislation and institutional requirements. Written informed consent for participation was not required for this study in accordance with the national legislation and the institutional requirements.

## Author Contributions

MM was the leader of the research group that conducted the study and organized the database. MM, NS, DS, MŽ, DA, GT, and VM contributed to the conception and design of the study. MM, NS, and DS performed the statistical analysis and wrote the first draft of the manuscript. MŽ, DA, GT, and VM reviewed and edited the first draft. All authors contributed to the article and approved the submitted version.

## Conflict of Interest

The authors declare that the research was conducted in the absence of any commercial or financial relationships that could be construed as a potential conflict of interest.

## Publisher’s Note

All claims expressed in this article are solely those of the authors and do not necessarily represent those of their affiliated organizations, or those of the publisher, the editors and the reviewers. Any product that may be evaluated in this article, or claim that may be made by its manufacturer, is not guaranteed or endorsed by the publisher.
